# A good response to glucocorticoid for Henoch–Schönlein purpura with abdominal pain and gastrointestinal bleeding in an adult: A CARE case report

**DOI:** 10.1097/MD.0000000000018602

**Published:** 2020-01-03

**Authors:** Fangfang Yi, Zhaohui Bai, Yingying Li, Xiangbo Xu, Xiaozhong Guo, Xingshun Qi

**Affiliations:** a Department of Gastroenterology, General Hospital of Northern Theater Command, Shenyang; b Postgraduate College, Dalian Medical University, Dalian; c Postgraduate College, Shenyang Pharmaceutical University, Shenyang; d Postgraduate College, Jinzhou Medical University, Jinzhou 121001, P.R. China.

**Keywords:** abdominal pain, adult, gastrointestinal bleeding, glucocorticoid, Henoch–Schönlein purpura

## Abstract

**Rationale:**

Henoch–Schönlein purpura (HSP) is a small-vessel vasculitis that has been extensively studied in children, but little is known about its natural history in adults. There is no consensus regarding the treatment of glucocorticosteroids use for HSP. The efficacy of glucocorticoid for preventing from severe complications or relapse is also controversial in HSP.

**Patient concerns:**

A 21-year-old male was admitted to the hospital due to abdominal pain for more than 20 days, hematochezia for more than 10 days, and rash for 2 days.

**Diagnoses:**

The diagnosis of HSP is based on the European League against Rheumatism and the Paediatric Rheumatology European Society in 2006.

**Interventions:**

The patient received glucocorticosteroids treatment for 17 days at the time of first hospitalization.

**Outcomes:**

The abdominal pain and hematochezia completely disappeared on the 6th day after the use of glucocorticosteroids, and purpura completely disappeared on the 8th day.

**Lessons:**

Our patient has a good response to glucocorticoid. Glucocorticosteroids may be effective for the treatment of HSP.

## 1 Introduction

Henoch–Schönlein purpura (HSP) is the most common systemic vasculitis in children.^[1]^ Annual incidence of HSP in children is estimated to be 15/100,000 cases.^[2]^ The proportion of males and females in children is close to 2:1.^[3]^ By comparison, it is more rare in adults with an estimated annual incidence of 1.3/100,000 cases.^[2]^ Compared with HSP in children, HSP in adults have more serious clinical manifestations and worse outcomes.^[4]^ Clinical characteristics of HSP include abdominal pain, gastrointestinal bleeding, nonthrombocytopenic palpable purpura, arthritis, and renal involvement.^[5]^ Most of them occur in autumn and winter, but its pathogenesis and causes are still unclear.^[6]^ In 2006, the diagnostic criteria published by the European League against Rheumatism and the Paediatric Rheumatology European Society which include palpable purpura in combination with at least one of other manifestations (ie, abdominal pain, immunoglobulin A deposition, arthritis or arthralgia, and renal involvement).^[7]^ There is no consensus on treatment of glucocorticoid for HSP in adults. The efficacy of glucocorticoid in preventing from severe complications or relapse is also controversial in HSP.^[8,9]^

In this case report, we showed that an adult patient with HSP presenting with abdominal pain and gastrointestinal bleeding had a good response by glucocorticoid.

## 2 Case presentation

On February 12, 2019, a 20-year-old male was admitted to our department due to abdominal pain for more than 20 days, hematochezia for more than 10 days, and rash for 2 days.

**Figure 1:**
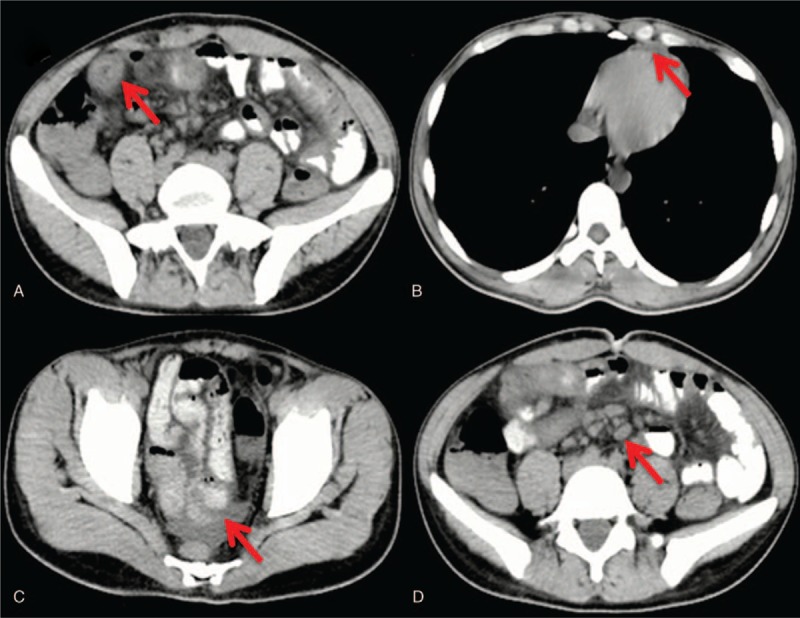
Chest and abdominal CT on February 11, 2019. Panel A, Localized thickening of small intestinal wall in the right lower abdomen (red arrow). Panel B, A small amount of pericardial effusion (red arrow). Panel C, Pelvic effusion (red arrow). Panel D, Abdominal lymph node enlargement (red arrow). CT = computed tomography.

His disease course was as follows. On January 23, 2019, the patient presented with sudden onset of abdominal pain with nausea, vomiting, and fatigue but without hematemesis or melena. On January 28, 2019, he received symptomatic treatment at his local hospital. On February 2, 2019, he had hematochezia which presented with dark red pasty stool 4 times a day with an amount of about 50 mL every time. On February 10, 2019, he developed diffuse purpura on his limbs which were not faded by pressing. He was treated with symptomatic treatment without glucocorticoid. On February 11, 2019, he was transferred to our hospital. Laboratory tests showed the followings with normal range in parentheses: white blood cell (WBC), 38.4 × 10^9^/L (3.5–9.5 × 10^9^/L); percentage of granulocyte (GR%), 89.9% (40–75%); platelet count (PLT), 465 × 10^9^/L (100–300 × 10^9^/L); gamma-glutamyl transpeptidase (GGT), 82.79 U/L (8–78 U/L); carbohydrate antigen 125, 74.76 U/mL (0–35 U/mL); and serum sodium, 132.0 mmol/L (137–147 mmol/L). Abdominal color Doppler ultrasound showed cholestasis and cholecystitis. Chest and abdominal computed tomography scans showed localized thickening of small intestinal wall in the right lower abdomen, a small amount of pericardial effusion, pelvic effusion, and abdominal lymph node enlargement (Fig. 1).

On February 12, 2019, he was admitted to our department. His previous medical history included haemorrhoidectomy. Physical examinations showed soft abdomen, deep tenderness in the middle abdomen without rebound pain, and diffuse purpuric rashes on his limbs. Laboratory tests showed WBC, 30.4 × 10^9^/L; GR%, 89.5%; red blood cell (RBC), 4.16 × 10^12^/L (4.3–5.8 × 10^12^/L); hemoglobin (Hb), 120 g/L (130–175 g/L); C-reactive protein (CRP), 25.68 mg/L (≤10 mg/L); hematocrit, 35.8% (40%–50%); and PLT, 327 × 10^9^/L. Fecal occult blood test was positive. Electrocardiogram showed sinus tachycardia. Therefore, according to the European League against Rheumatism and the Paediatric Rheumatology European Society criteria in 2006, HSP was diagnosed. He was initially treated with somatostatin 3000 μg and pantoprazole sodium 40 mg.

On February 13, 2019, the patient had dark red bloody stool twice again with the total amount of about 400 mL. His heart rate was about 125 beats per minute. Laboratory tests showed WBC, 34.3 × 10^9^/L; GR%, 88.5%; RBC, 4.66 × 10^12^/L; Hb, 135 g/L; erythrocyte sedimentation rate (ESR), 22 mm/h (0–15 mm/h); CRP, 39 mg/L; PLT, 403 × 10^9^/L; albumin, 23.1 g/L (40–55 g/L); serum calcium (Ca), 1.97 mmol/L (2.08–2.6 mmol/L); procalcitonin, 0.281 ng/mL (0–0.05 ng/mL); D-dimer, 24.11 mg/L; immunoglobulin A, 3.20 g/L (0.7–4 g/L); immunoglobulin G, 8.47 g/L (7–16 g/L); immunoglobulin M, 0.37 g/L (0.4–2.3 g/L); complement C3, 1.081 g/L (0.75–1.55 g/L); and complement C4, 0.185 g/L (0.1–0.4 g/L). Hepatitis B and C virus were negative. A dermatologist's consultation suggested discontinuation of suspected drugs, oral use of desloratadine 5 mg/d, ebastine 20 mg/d, and melilotus extract tablets 3600 mg/d, external use of calamine lotion 6 times a day, intravenous infusion of compound glycyrrhizin 60 mL/d, and glucocorticoid therapy if appropriate. Octreotide was given intravenously with a dosage of 0.3 mg every 12 hours. Levofloxacin was given intravenously with a dosage of 0.5 g/d. Calcium gluconate was given intravenously with a dosage of 10 mL/d. Human albumin was given intravenously with a dosage of 10 g.

On February 14, 2019, the patient had hematochezia twice with the total amount of about 600 mL. Laboratory tests showed WBC, 39.2 × 10^9^/L; GR%, 91.0%; PLT, 408 × 10^9^/L; and Hb, 138 g/L. Considering that gastrointestinal bleeding episodes persisted, glucocorticoid therapy was given after the informed consent written by his father. Methylprednisolone was given intravenously with a dosage of 80 mg twice a day.

On February 15, 2019, his abdominal pain was improved obviously and heart rate was dropped to normal with an average of 80 beats per minute. He intermittently had hematochezia with the total amount of about 400 mL. Laboratory tests showed WBC, 32.2 × 10^9^/L; GR%, 90.1%; RBC, 3.75 × 10^12^/L; Hb, 112 g/L; ESR, 27 mm/h; Ca, 1.94 mmol/L; and Streptolysin O, 238.1 IU/mL (0–20 IU/mL). Antinuclear antibody and fecal parasites examinations were negative. On February 16, 2019, the dosage of methylprednisolone was reduced to 40 mg twice a day.

On February 18, 2019, laboratory tests showed WBC, 21.0 × 10^9^/L; GR%, 77.1%; RBC, 3.93 × 10^12^/L; Hb, 118 g/L; and ESR, 13 mm/h. On February 19, 2019, his abdominal pain and hematochezia disappeared. Oral methylprednisolone was employed with a dosage of 20 mg twice a day. Subcutaneous injection of octreotide was given with a dosage of 0.1 mg every 8 hours.

**Figure 2:**
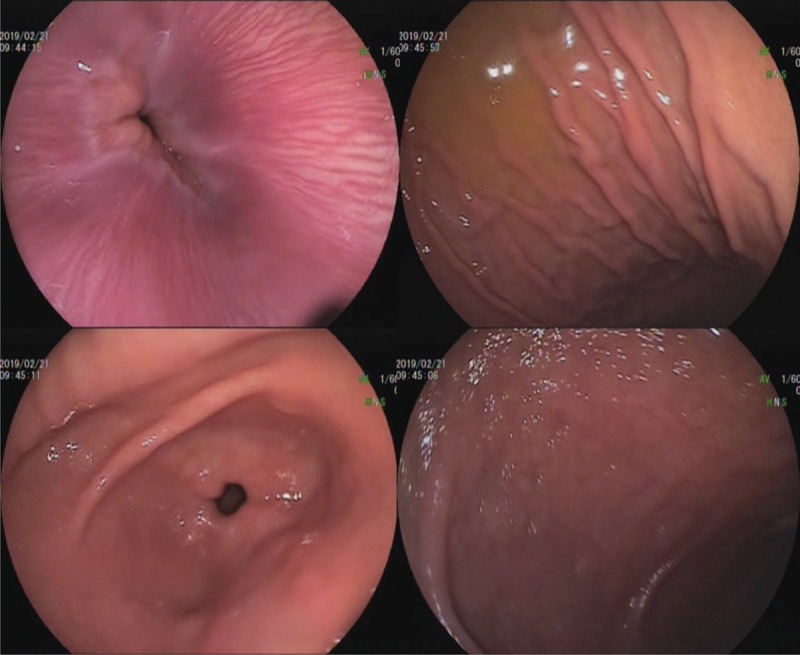
Gastroscopy on February 21, 2019 showing chronic superficial gastritis with bile reflux.

On February 21, 2019, his condition was relatively stable. Gastroscopy showed chronic superficial gastritis with bile reflux (Fig. 2). The patient's rash disappeared after glucocorticoid treatment. The dosage of methylprednisolone was gradually decreased.

On March 1, 2019, laboratory tests showed WBC, 11.3 × 10^9^/L; GR%, 72.8%; RBC, 3.69 × 10^12^/L; Hb, 110 g/L; ESR, 8 mm/h; CRP, 0.50 mg/L; and GGT, 56.73 U/L. On March 3, 2019, the patient was in a good condition without abdominal pain or hematochezia. Methylprednisolone was discontinued. On March 5, 2019, he was discharged.

On March 13, 2019, the patient was re-admitted to our department due to recurrence of abdominal pain, which developed after cough, sputum, and runny nose. Laboratory tests showed WBC, 12.15 × 10^9^/L; GR%, 80.1%; and ESR, 37.6 mm/h. Fecal occult blood test was positive. Routine urine test showed that urinary occult blood and protein were positive. On March 16, 2019, methylprednisolone was given intravenously with a dosage of 60 mg/day. Esomeprazole was given intravenously with a dosage of 40 mg/d. On April 1, 2019, his abdominal pain improved significantly. Laboratory tests showed WBC, 13.0 × 10^9^/L; GR%, 76.2%; and ESR, 16 mm/h. Fecal occult blood test and routine urine test were negative. On April 2, 2019, he was discharged and took a stepwise reduction of dosage of oral methylprednisolone.

## 3 Discussion

### 3.1 Effectiveness of glucocorticoid

Currently, the evidence about the application of glucocorticoid for HSP is insufficient. Some researchers reported that early use of prednisone in HSP did not reduce either the risk of renal involvement within one year or the risk of acute gastrointestinal complications.^[8]^ In contrast, Ronkainen et al found that the mean duration of total days abdominal pain was shortened by 1.2 days in the prednisone group.^[10]^ Similarly, Dr Saulsbury also reported that glucocorticoids could accelerate the relief of abdominal pain.^[11]^

Some authors suggested that the dose of glucocorticoid was 1 to 2 mg/kg/d and gradually reduced in the treatment of HSP.^[10,11]^ Our patient's weight is 60 kg, and intravenous glucocorticoid was given at a dose of 80 mg twice a day for the first 3 days, followed by a dose of 40 mg twice a day for the second 3 days. Then, the glucocorticoid was taken orally and gradually reduced until he was discharged.

**Figure 3:**
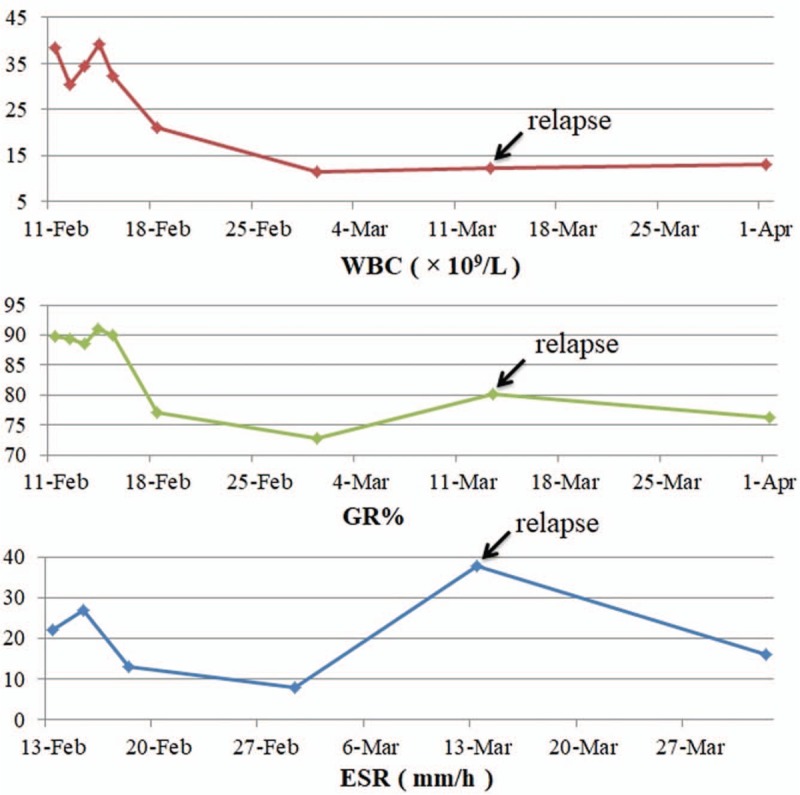
Changes of WBC, GR%, and ESR during hospitalizations. ESR = erythrocyte sedimentation rate, GR% = percentage of granulocyte, WBC = white blood cell.

In our patient, the abdominal pain relieved since the first use of glucocorticoids. Then, the rash completely disappeared on the 8th day after the first use of glucocorticoids. The laboratory parameters including WBC, GR%, and ESR gradually decreased to normal (Fig. 3). Abdominal pain is a common clinical manifestation in patients with HSP. Up to 75% of HSP patients have gastrointestinal tract involvement, which is often characterized by diffuse colic pain.^[3]^

In addition, HSP is considered a self-limiting disease with resolution of symptoms within 4 to 6 weeks of onset, but can lead to some poor outcomes, such as gastrointestinal bleeding, intussusception, and end-stage renal disease.^[9]^ Occult or symptomatic gastrointestinal bleeding, such as hematemesis, melena, and rectal bleeding, is observed in 50% of children with HSP.^[12]^ By comparison, gastrointestinal bleeding associated with HSP is rare in adults, accounting for only 4%.^[13]^ The outcomes of gastrointestinal bleeding are poor, and some patients require blood transfusion or surgery, and even die.^[14]^ A case report suggested that methylprednisolone pulse treatment might be effective for gastrointestinal bleeding in HSP.^[15]^ In our case, the use of glucocorticoid therapy in the patient with gastrointestinal bleeding is effective.

### 3.2 Relapse and its risk factors

Our case developed abdominal pain again accompanied by hematuria and proteinuria without hematochezia or rash about 1 week after discharge. Abdominal pain is the most common presentation for disease recurrence (about 63%).^[12]^ Hematuria and proteinuria indicating renal involvement are more common in adults than in children^[2]^; and 67% of patients develop hematuria at the 5th week of onset in adults.^[16]^

The precipitating factor for relapse in our case might be a recent history of infection, which presented with cough, sputum, and runny nose with an increased ESR level on laboratory tests (Fig. 3). The upper respiratory tract infection is a potential trigger in HSP.^[11]^ Researchers also suggested that the relapse was strongly associated with a high ESR value.^[17]^

In conclusion, our patient with HSP had a good response to glucocorticoid therapy. However, current evidence in regard to the efficacy of glucocorticoid or relapse is lacking. Further researches are necessary to explore the relationship between the use of glucocorticoid and prognosis of HSP.

## Author contributions

**Conceptualization:** Xingshun Qi.

**Formal analysis:** Xiaozhong Guo, Xingshun Qi.

**Methodology:** Fangfang Yi, Zhaohui Bai, Yingying Li.

**Supervision:** Zhaohui Bai, Xiangbo Xu, Xingshun Qi.

**Writing – original draft:** Fangfang Yi.

**Writing – review and editing:** Xingshun Qi, Xiangbo Xu Zhaohui Bai.

Xingshun Qi orcid: 0000-0002-9448-6739.
